# The *Aspergillus fumigatus* Secretome Alters the Proteome of *Pseudomonas aeruginosa* to Stimulate Bacterial Growth: Implications for Co-infection

**DOI:** 10.1074/mcp.RA120.002059

**Published:** 2020-11-23

**Authors:** Anatte Margalit, James C. Carolan, David Sheehan, Kevin Kavanagh

**Affiliations:** Department of Biology, Maynooth University, Maynooth, Co. Kildare, Ireland

**Keywords:** Bacteria, pathogens, secretome, cell biology, proliferation, Aspergillus, cystic fibrosis, Pseudomonas

## Abstract

Individuals with cystic fibrosis are susceptible to co-infection by *Aspergillus fumigatus* and *Pseudomonas aeruginosa*. Despite the persistence of *A. fumigatus* in the cystic fibrosis lung *P. aeruginosa* eventually predominates as the primary pathogen. Several factors are likely to facilitate *P. aeruginosa* colonization in the airways, including alterations to the microbial environment. The cystic fibrosis airways are hypoxic, nitrate-rich environments, and the sputum has higher amino acid concentrations than normal. In this study, significant growth proliferation was observed in *P. aeruginosa* when the bacteria were exposed to *A. fumigatus* culture filtrates (CuF) containing a high nitrate content. Proteomic analysis of the *A. fumigatus* CuF identified a significant number of environment-altering proteases and peptidases. The molecular mechanisms promoting bacterial growth were investigated using label-free quantitative (LFQ) proteomics to compare the proteome of *P. aeruginosa* grown in the *A. fumigatus* CuF and in CuF produced by a *P. aeruginosa-A. fumigatus* co-culture, to that cultured in *P. aeruginosa* CuF. LFQ proteomics revealed distinct changes in the proteome of *P. aeruginosa* when cultured in the different CuFs, including increases in the levels of proteins involved in denitrification, stress response, replication, amino acid metabolism and efflux pumps, and a down-regulation of pathways involving ABC transporters. These findings offer novel insights into the complex dynamics that exist between *P. aeruginosa* and *A. fumigatus*. Understanding the molecular strategies that enable *P. aeruginosa* to predominate in an environment where *A. fumigatus* exists is important in the context of therapeutic development to target this pathogen.

## Teaser

The growth *of Pseudomonas aeruginosa* increased exponentially when exposed to the culture filtrates produced by *Aspergillus fumigatus* and by co-cultures of *A. fumigatus* and *P. aeruginosa* in a nutrient-poor, nitrate-rich medium. Qualitative proteomic analysis of the *A. fumigatus* culture filtrates identified several secreted proteases and peptidases, including known human allergens. LFQ proteomics performed on *P. aeruginosa* exposed to the culture filtrates identified changes in several pathways and processes including an increase in outer-membrane proteins and stress response proteins.

## Graphical Abstract

**Figure:**
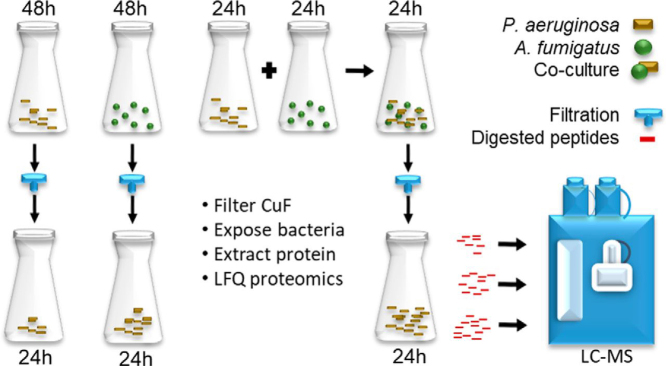

• *Pseudomonas aeruginosa* growth increases in *Aspergillus fumigatus* culture filtrates.
• *A. fumigatus* culture filtrates are characterized by a range of peptidases and proteases.
• LFQ proteomics characterizes the response of *P. aeruginosa* to *A. fumigatus* culture filtrates.
• *A. fumigatus* creates an environment for *P. aeruginosa* to proliferate.

The airways of cystic fibrosis patients are chronically colonized by a diverse range of microbial pathogens, the composition of which changes throughout life (1, 2). Alteration to the pulmonary environment caused by inter-microbial interactions and pathogen-host interactions influence the type of microbes that can engage in sustained infection (3). The opportunistic bacterial pathogen *Pseudomonas aeruginosa* is the primary cause of morbidity and mortality among individuals with cystic fibrosis and it is estimated that 60–80% of cystic fibrosis patients experience chronic *P. aeruginosa* infection by the age of 20 years (4, 5). *P. aeruginosa* owes its success as a human pathogen to its relatively large genome (∼6.3 Mbp), which encodes a variety of virulence factors and regulatory proteins that enable this bacterium to rapidly adapt to, and colonize multiple niches, including the hypoxic environment of the cystic fibrosis lung (6, 7).

*Aspergillus fumigatus* is the most prevalent fungal pathogen isolated from cystic fibrosis airways, affecting up to 57% of patients (8, 9, 10). It is the causative agent of allergic bronchopulmonary aspergillosis (ABPA), a hypersensitivity disorder resulting from the inhalation of fungal conidia. Although co-colonization of the cystic fibrosis airways by *P. aeruginosa* and *A. fumigatus* is rare (3.1–15.8%) disease prognosis is poor when both are present (8, 11). However, sequential infection with *A. fumigatus* and *P. aeruginosa* is more common and despite the prevalence and persistence of *A. fumigatus* in younger cystic fibrosis patients (12), *P. aeruginosa* predominates as the primary pathogen from the second decade of life. This suggests that interactions with other pathogens such as *A. fumigatus* may influence the pathogenicity of *P. aeruginosa* by altering its virulence, for example by increasing the production of elastases (13).

*P. aeruginosa* produces a range of products that inhibit *A. fumigatus* growth including pyoverdin, an iron chelator that deprives *A. fumigatus* of iron from the environment thereby inhibiting fungal biofilm formation (14). *A. fumigatus* counteracts the effects of pyoverdin with its own siderophores which cannot be exploited by *P. aeruginosa* (15). *P. aeruginosa* phenazines, Phenazine-1-carboxylic acid and 1-hydroxyphenazine (1-HP), inhibit growth of *A. fumigatus* by inducing reactive oxygen species (ROS) production and chelating iron, respectively (16). The accumulation of ROS may inhibit *A. fumigatus* biofilm by mediating apoptosis in the fungal biofilm (17). Although direct contact with *P. aeruginosa* has an inhibitory effect on *A. fumigatus* growth, volatile organic compounds released by the bacteria are believed to stimulate the growth of *A. fumigatus* (18, 19). *A. fumigatus* may also influence *P. aeruginosa* growth through the production of gliotoxin, a secondary metabolite with anti-bacterial activity (20).

We report here the changes that occur in the growth and to the proteome of *P. aeruginosa* when exposed to the culture filtrate produced by *A. fumigatus*. It was hypothesized that *A. fumigatus* creates an environment that promotes a metabolic-driven increase in *P. aeruginosa* growth that results in it outcompeting the fungus. Qualitative analysis using mass spectrometry and RP-HPLC was performed to analyze this environment and to characterize the contents of the *A. fumigatus* culture filtrate. Label-free quantitative (LFQ) proteomics was employed to characterize changes to the proteome of *P. aeruginosa* when exposed to the culture filtrate of (1) *A. fumigatus* alone (2) the culture filtrate of an *A. fumigatus-P. aeruginosa* co-culture and (3) the culture filtrate of *P. aeruginosa* alone. The results offer novel insights into the biological pathways and processes that enable *P. aeruginosa* to outcompete *A. fumigatus* and hence predominate as the primary pathogen under these conditions.

## EXPERIMENTAL PROCEDURES

##### Experimental Design and Statistical Rationale

The effect of culture filtrates (CuF) produced in Czapek-Dox liquid media by (1) *P. aeruginosa* (control), (2) *A. fumigatus* or (3) *A. fumigatus* - *P. aeruginosa* co-cultures (where 24-hour cultures of *A. fumigatus* and *P. aeruginosa* were co-incubated for a further 24 h) on *P. aeruginosa* growth was measured by determining the optical density (OD_600_) of bacterial cultures. The concentration of gliotoxin produced by *A. fumigatus* ATCC 26933 and Δ*gliZ* in Czapek-Dox at different time-points was detected by RP-HPLC. The amino acid concentration in the culture filtrates, was determined by the ninhydrin assay and measured against a standard curve generated using serine and glutamine. Specific amino acids were detected by RP-HPLC post-derivitization of hydrolyzed culture filtrates. Four biological replicates were used in growth experiments, gliotoxin analysis and amino acid tests. Bacterial growth was analyzed using GraphPad Prism 5. One-way ANOVA with Tukey's multiple comparisons test was performed to compare the growth of bacteria in different culture filtrates and the differences in gliotoxin concentrations. Two-sample t-tests were performed on comparisons between bacterial cultures exposed to Czapek-Dox un-supplemented and supplemented with amino acids. *p* values <0.05 were considered significant.

The changes that occur to the *P. aeruginosa* proteome resulting from exposure to CuFs produced in Czapek-Dox were detected by LFQ proteomics. To validate the biological reliability of measurements, proteomic analysis was performed on four biological replicates of bacteria grown in the different CuF. For global proteomic analysis of the bacterial response to CuFs produced in Czapek-Dox, proteins that were identified in at least four out of four replicates were used for comparison. Student's t-tests (pairwise comparisons) were performed for comparisons between bacteria cultured in *A. fumigatus* CuF and *P. aeruginosa* CuF, Co-culture CuF and *P. aeruginosa* CuF and Co-culture CuF and *A. fumigatus* CuF, with 1% false discovery rate (FDR) cut-off for all data sets. Analysis of variance (ANOVA) was performed for multiple samples across all four groups using a permutation-based FDR of 5% and below to indicate statistically significant differentially abundant (SSDA) proteins. Enrichment analysis was performed on the Gene Ontology terms for the major protein clusters identified by hierarchical clustering using a Fisher's exact test (Benjamini-Hochberg corrected FDR of 4%).

##### Preparation of Aspergillus fumigatus Culture Filtrate

*A. fumigatus* (ATCC 26933) and a gliotoxin-deficient strain of *A. fumigatus* Af293 (Δ*gliZ*) were grown and maintained on Sabouraud dextrose agar (Oxoid) at 37 °C. Conidia were harvested using 0.01% Tween-80 and washed twice in phosphate buffered saline (PBS). Conidial number was ascertained by hemocytometry and conidia were added to Czapek-Dox liquid media (Duchefa) at a final concentration of 5 × 10^5^/ml. The cultures were incubated for 24, 48, 72, and 96 h at 37 °C in an orbital incubator, at 200 *rpm*. After each time point, the hyphal mass was harvested, and the wet weight was recorded. The *A. fumigatus* culture-filtrates (CuF) were filter-sterilized using 0.2 μm filtropur S filters (Sarstedt).

##### Extraction of Gliotoxin from A. fumigatus Culture Filtrate

The CuF of *A. fumigatus* ATCC 26933 type and Δ*gliZ* strains were mixed with an equal volume (20 ml) chloroform (Fisher Scientific) and mixed for 2 h. The chloroform fraction was collected and dried by rotary evaporation in a Büchi rotor evaporator (Brinkmann Instruments; Westbury, NY). Dried extracts were dissolved in 500 μl HPLC grade methanol (Fisher Scientific) and stored at −20 °C.

##### Quantification of Gliotoxin by RP-HPLC

Gliotoxin was detected by Reversed Phase-HPLC (Shimadzu). The mobile phase was 34.9% (v/v) acetonitrile (Fisher Scientific), 0.1% (v/v) trifluoroacetic acid (TFA) (Sigma Aldrich) and 65% (v/v) HPLC grade deionized water (ddH_2_O). Gliotoxin extract (20 μl) (Sigma Aldrich) was injected onto a Lunar Omega, 5 μm polar C18, LC column (Phenomenex). Peaks were visualized at 254 nm. A standard curve of peak area *versus* gliotoxin concentration was constructed using gliotoxin standards (0.1, 0.25, 0.5, 1.0 μg/10 μl) dissolved in methanol.

##### Effect of A. fumigatus CuF on P. aeruginosa Growth

*P. aeruginosa* (PAO1) was cultured overnight in nutrient broth (Oxoid). Bacterial suspension (100 μl, OD_600_ 0.1)* was added to each well of a 96-well plate (Corning). Czapek-Dox (100 μl) or 100 μl of the 24-, 48-, 72-, and 96-hour *A. fumigatus* CuF was added to each row. The plate was incubated at 37 °C for 24 h and read in a plate reader (Bio-Tek Synergy HT) at 600 nm. *OD 1.0 (600 nm) equates to ∼3 × 10^8^ CFU/ml.

##### Preparation of Pseudomonas aeruginosa Culture Filtrate

*P. aeruginosa* was cultivated on nutrient agar (Oxoid) at 37 °C. A sterile loop was used to inoculate Czapek-Dox liquid media with the bacteria. Bacterial cultures were incubated for 48 h at 37 °C in an orbital incubator, at 200 *rpm*. The concentration of the bacterial suspension was quantified by obtaining the optical density at 600 nm (OD_600_). Bacterial densities were measured after 48 h incubation (OD 0.19 ± 0.022). Bacteria were separated from the supernatant by centrifugation (15 min, 2000 *g*). *P. aeruginosa* culture filtrates were sterilized using 0.2 μm filtropur S filters (Sarstedt) and are hereafter referred to as *P. aeruginosa* CuF.

##### Preparation of Co-culture Culture Filtrate

*A. fumigatus* conidia (5 × 10^5^ conidia/ml) and *P. aeruginosa* (loopful) were cultured in Czapek-Dox liquid media for 24 h at 37 °C in an orbital incubator at 200 *rpm*. Prior to co-incubation with *A. fumigatus* cultures, *P. aeruginosa* density was determined to ensure similar densities across the groups (OD_600_ 0.2 ± 0.0087). The co-culture was incubated under the same conditions for a further 24 h to give a total incubation period of 48 h for each pathogen to match the total incubation time applied for the fungal- and bacterial-only cultures. The hyphae were removed from the culture and the bacteria were removed by centrifugation (20 min at 2000 *g*). The Co-culture culture filtrates were sterilized using 0.2 μm filtropur S filters (Sarstedt) and are hereafter referred to as Co-culture CuF. *A. fumigatus* CuF, *P. aeruginosa* CuF and Co-culture CuF were also produced as described above in minimal media (MM) and synthetic cystic fibrosis medium (SCFM) (21).

##### Analysis of P. aeruginosa Growth in Czapek-Dox Media

Czapek-Doz liquid media was inoculated with *P. aeruginosa* using a sterile loop and incubated at 37 °C in an orbital incubator. The growth of the bacterial suspension was measured at 4, 18, 24, 48, 72, and 96 h by obtaining the optical density at 600 nm (OD_600_).

##### Analysis of Amino Acid Concentration Culture Filtrates

Culture filtrates from *A. fumigatus*, *P. aeruginosa* and Co-cultures were analyzed for amino acids using 2% ninhydrin (Sigma) dissolved in ethanol (22). *A. fumigatus* 48-hour CuF and sterile Czapek-Dox media were hydrolyzed with HCl (6 m) by microwave (CEM Discoverer Microwave Synthesizer) for one hour at 110^°^C (23). Amino acid standards (Amino acid standard H, (Pierce^TM^), Czapek-Dox media and CuF were PITC-derivitized according to the manufacturer's instructions (Pierce^TM^). Amino acid identification was performed by RP-HPLC under the same gradient conditions as for the gliotoxin analysis and visualized at 254 nm.

##### Exposure of P. aeruginosa to A. fumigatus CuF, P. aeruginosa CuF, Co-culture CuF or Amino Acids

*P. aeruginosa* was cultured in Czapek-Dox, MM or SCFM (25 ml) for 24 h, to stationary phase. Bacterial density (OD_600_) was determined to ensure equal densities across all groups. Where necessary, bacterial culture densities were adjusted to give a final value of OD 0.1 in 25 ml prior to incubation with culture filtrates produced by *A. fumigatus*, *P. aeruginosa* or the co-cultures in corresponding growth media (50 ml). To investigate the effect of amino acids on bacterial growth, *P. aeruginosa* suspensions cultured in Czapek-Dox for 24 h (25 ml, adjusted to OD.0.1) were exposed to sterile Czapek-Dox (50 ml) or Czapek-Dox supplemented with amino acids; aspartic acid (2.37 × 10^5^ pmol/ml), glutamic acid (8.99 × 10^5^ pmol/ml), serine (8.82 × 10^5^ pmol/ml), threonine (8.93 × 10^5^ pmol/ml), methionine (1.17 × 10^6^ pmol/ml), valine (1.14 × 10^5^ pmol/ml) and leucine (1.58 × 10^5^ pmol/ml). Bacterial cultures were incubated for a further 24 h at 37 °C in an orbital incubator, at 200 *rpm.* Growth of the over-night cultures were measured by obtaining the optical density at 600 nm prior to, and post exposure to the supplemented liquid media.

##### Preparation of A. fumigatus CuF and P. aeruginosa Proteins for Mass Spectrometry A. fumigatus CuF Proteins

Proteins from 48-hour *A. fumigatus* CuF produced in Czapek-Dox were concentrated by centrifugation (Vivaspin 20, 3kDa MWCO PES, Sartorius). Protein was quantified using the Qubit^TM^ quantification system (Invitrogen), following the manufacturer's instructions and 50 μg of protein was precipitated overnight in acetone at −20 °C.

##### P. aeruginosa Cultured in A. fumigatus CuF, Co-culture CuF or P. aeruginosa CuF

Bacteria were separated from the culture filtrate by centrifugation (15 min, 2000 × *g*) and the resulting bacterial pellet was washed twice with PBS. *Ex situ* cell lysis was performed by re-suspending the bacterial pellet in 1 ml cell lysis buffer (8 m urea, 2 m thiourea, and 0.1 m Tris-HCl (pH 8.0) dissolved in ddH_2_O), supplemented with protease inhibitors (aprotinin, leupeptin, pepstatin A, and N-a-tosyl-l-lysine chloromethyl ketone hydrochloride (TLCK) (10 μg/ml) and phenylmethylsulfonyl fluoride (PMSF) (1 mm/ml). Cell lysates were sonicated with a sonication probe (Bendelin Senopuls), three times for 10 s at 50% power. The cell lysate was subjected to centrifugation (Eppendorf Centrifuge 5418) for 8 mins at 14,500 × *g* to pellet cellular debris. The supernatant was removed and quantified using the Bradford method. Each sample (100 μg) was subjected to overnight acetone precipitation.

##### Label Free Mass Spectrometry (LF/MS)

*A. fumigatus* CuF protein and *P. aeruginosa* proteins were pelleted by centrifugation for 10 min at 14,500 × *g.* The acetone was removed, and the protein pellet was re-suspended in 25 μl sample resuspension buffer (8 m urea, 2 m thiourea, 0.1 m Tris-HCl (pH 8.0) dissolved in ddH_2_O). An aliquot (2 μl) was removed from each sample and quantified using the Qubit^TM^ quantification system (Invitrogen), following the manufacturer's instructions. Ammonium bicarbonate (125 μl, 50 mm) was added to the remaining 20 μl of each sample. The protein sample was reduced by adding 1 μl 0.5 m dithiothreitol (DTT) and incubated at 56 °C for 20 min, followed by alkylation with 0.55 m iodoacetamide at room temperature, in the dark for 15 min. Protease Max Surfactant Trypsin Enhancer (Promega) (1 μl, 1% w/v) and Sequence Grade Trypsin (Promega) (0.5 μg/μl) was added to the proteins and incubated at 37 °C for 18 h. Digestion was terminated by adding TFA (1 μl, 100%) to each tryptic digest sample, and incubated at room temperature for 5 mins. Samples were centrifuged for 10 min at 14,500 × *g* and purified for mass spectrometry using C18 Spin Columns (Pierce), following the manufacturer's instructions. The eluted peptides were dried in a SpeedyVac concentrator (Thermo Scientific Savant DNA120) and resuspended in 2% *v/v* acetonitrile and 0.05% *v/v* TFA to give a final peptide concentration of 1 μg/μl. The samples were sonicated for 5 mins to aid peptide resuspension, followed by centrifugation for 5 mins at 14,500 × *g.* The supernatant was removed and used for mass spectrometry. Four independent biological replicates for each group were analyzed in this study.

##### Mass Spectrometry: LC/MS Xcalibur Instrument Parameters for P. aeruginosa Proteomic Data Acquisition

Digested proteins (500 ng) isolated from *A. fumigatus* CuF and 750 ng of each digested *P. aeruginosa* protein sample were loaded onto a QExactive (ThermoFisher Scientific) high-resolution accurate mass spectrometer connected to a Dionex Ultimate 3000 (RSLCnano) chromatography system. Peptides were separated by an increasing acetonitrile gradient on a 50 cm EASY-Spray PepMap C18 column with 75 μm diameter (2 μm particle size), using a 65 min reverse phase gradient for the *A. fumigatus* CuF and a 133 min reverse phase gradient for the *P. aeruginosa* proteins at a flow rate of 300 nL/min^−1^. All data were acquired with the mass spectrometer operating in an automatic dependent switching mode. A full MS scan at 70,000 resolution and a range of 400–1600 *m/z*, was followed by an MS/MS scan at 17,500 resolution, with a range of 200–2000 *m/z* to select the 15 most intense ions prior to MS/MS.

Qualitative analysis of the proteome arising from the *A. fumigatus* CuF was investigated using Proteome Discoverer 1.4 and Sequest HT (SEQUEST HT algorithm, Thermo Scientific). Identified proteins were searched against the UniProtKB database (*Neosartorya fumigata*; downloaded 18/05/2018; 9647 entries). Search parameters applied for protein identification were as follows: (1) enzyme name - trypsin, (2) an allowance of up to two missed cleavages, (3) peptide mass tolerance set to 10 ppm, (4) MS/MS mass tolerance set to 0.02 Da, (5) carbamidomethylation set as a fixed modification and (6) methionine oxidation set as a variable modification (24). Peptide probability was set to high confidence (with an FDR ≤ 0.01% as determined by Percolator validation in Proteome Discoverer). Peptides with minimum score of two and fewer than three unique peptides were excluded from further analysis. The MS proteomics data and Proteome Discoverer search output files have been deposited to the ProteomeXchange Consortium (25) via the PRIDE partner repository with the data set identifier PXD018112.

Quantitative analysis (protein quantification and LFQ normalization of the MS/MS data) of the *P. aeruginosa* proteome arising from exposure to the different CuFs, was performed using MaxQuant version 1.5.3.3 (http://www.maxquant.org) following the general procedures and settings outlined in (26). The Andromeda search algorithm incorporated in the MaxQuant software was used to correlate MS/MS data against the Uniprot-SWISS-PROT database for *P. aeruginosa* PAO1 and *A. fumigatus* Af293 (downloaded 11/09/2018; 15286 entries). The following search parameters were used: first search peptide tolerance of 20 ppm, second search peptide tolerance 4.5 ppm with cysteine carbamidomethylation as a fixed modification and N-acetylation of protein and oxidation of methionine as variable modifications and a maximum of two missed cleavage sites allowed. False discovery rate (FDR) was set to 1% for both peptides and proteins, and the FDR was estimated following searches against a target-decoy database. Peptides with minimum length of seven amino acid length were considered for identification and proteins were only considered identified when observed in three replicates of one sample group.

##### Data Analysis of the P. aeruginosa Proteome

Perseus v.1.5.5.3 (www.maxquant.org/) was used for data analysis, processing and visualization. Normalized LFQ intensity values were used as the quantitative measurement of protein abundance for subsequent analysis. The data matrix was first filtered for the removal of contaminants and peptides identified by site. LFQ intensity values were log_2_ transformed and each sample was assigned to its corresponding group, *i.e. P. aeruginosa* exposed to *P. aeruginosa* CuF (control) *versus P. aeruginosa* exposed to *A. fumigatus* CuF or Co-culture CuF. Proteins not found in four out of four replicates in at least one group were omitted from the analysis. A data-imputation step was conducted to replace missing values with values that simulate signals of low abundant proteins chosen randomly from a distribution specified by a downshift of 2 times the mean standard deviation (S.D.) of all measured values and a width of 0.3 times this S.D.

Normalized LFQ intensity values were used for a principal component analysis (PCA). Exclusively expressed proteins (those that were uniquely expressed or completely absent in one group) were identified from the pre-imputation data set and included in subsequent analyses. Gene ontology (GO) mapping was also performed in Perseus using the UniProt gene ID for all identified proteins to query the Perseus annotation file (downloaded September 2018) and extract terms for gene ontology biological process (GOBP), gene ontology cellular component (GOCC), gene ontology molecular function (GOMF) and Kyoto Encyclopedia of Genes and Genomes (KEGG) name.

To visualize differences between two samples, pairwise Student's t-tests were performed for all using a cut-off of *p* < 0.05 on the post-imputated data set. Volcano plots were generated in Perseus by plotting negative log *p* values on the *y* axis and log_2_ fold-change values on the *x* axis for each pairwise comparison. The ‘categories’ function in Perseus was utilized to highlight and visualize the distribution of various pathways and processes on selected volcano plots. Statistically significant (ANOVA, *p* < 0.05) and differentially abundant proteins (SSDA), *i.e.* with relative fold change of plus or minus 2.0 were chosen for further analysis. The log_2_ transformed LFQ intensities for all SSDA proteins were Z-score normalized and used for hierarchical clustering of samples and SSDA proteins using Euclidean distance and average linkage.

GO and KEGG term enrichment analysis was performed on the major protein clusters identified by hierarchical clustering using a Fisher's exact test (a Benjamini-Hochberg corrected FDR of 4%) for enrichment in Uniprot Keywords, GOBP, GOCC, GOMF and KEGG (FDR <4%). The MS proteomics data and MaxQuant search output files have been deposited to the ProteomeXchange Consortium (25) via the PRIDE partner repository with the data set identifier PXD015056.

## RESULTS

##### Analysis of the Effect of A. fumigatus culture filtrate on P. aeruginosa growth

**Fig. 1:**
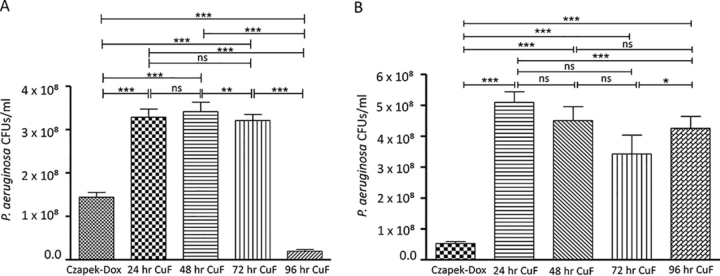
*A*, Changes in growth of a 24-hour *P. aeruginosa* culture incubated with sterile Czapek-Dox media (control) or 24-hour, 48-hour, 72-hour or 96-hour *A. fumigatus* wild-type CuF for 24 h. Maximum growth increase was observed in bacteria exposed to the 48-hour CuF and growth inhibition was observed in bacteria incubated with 96-hour CuF. *B*, Changes in growth of a 24-hour *P. aeruginosa* culture incubated in sterile Czapek-Dox media, or 24-hour, 48-hour, 72-hour or 96-hour *A. fumigatus* Δ*giZ* CuF for 24 h. Growth was not inhibited by the 96-hour Δ*gliZ* CuF.

The ability of *P. aeruginosa* to grow in Czapek-Dox, a sucrose-based medium with sodium nitrate as the sole nitrogen source was investigated. *P. aeruginosa* growth increased in a time-dependent manner until reaching stationary phase at 48 h (supplemental Fig. S1). To examine the effect of the *A. fumigatus* secretome produced in this media on the growth of *P. aeruginosa*, *A. fumigatus* culture filtrates (CuFs) were isolated from cultures grown in Czapek-Dox for 24, 48, 72, and 96 h. Toxicity assays revealed that the 24-, 48-, and 72-hour *A. fumigatus* CuFs had a growth promoting effect on *P. aeruginosa* compared with the control (sterile Czapek-Dox), but supernatants from the 96-hour CuF had a growth inhibiting effect (Fig. 1*A*). Czapek-Dox is frequently used with *A. fumigatus* as a growth medium for the production of gliotoxin. Because of this, the levels of gliotoxin in the *A. fumigatus* CuFs used for the toxicity assays here were quantified by RP-HPLC. The highest concentration of gliotoxin (2.29 μg ±0.06/mg hyphae, *p* < 0.05) was detected in the 96 h CuF (supplemental Fig. S2A) and correlated with the growth inhibition of *P. aeruginosa* in Fig. 1*A*. To confirm that gliotoxin was causing the growth inhibitory effect, a gliotoxin-deficient strain of *A. fumigatus*, which lacks the GliZ gene responsible for gliotoxin production, was cultured for 24, 48, 72, and 96 h. Gliotoxin deficiency was confirmed by RP-HPLC (supplemental Fig. S2B). *P. aeruginosa* was exposed to the CuFs produced by the Δ*gliZ* mutant. Bacterial growth was somewhat greater when exposed to the CuFs produced by the Δ*gliZ* strain at all time-points compared with that where bacteria were exposed to CuFs produced by the wild-type strain (Fig. 1*A* and 1*B*). Differences in the parental origin of the strains may account for the variation in growth rate. Bacterial growth in cultures exposed to the 96-hour CuF produced by the Δ*gliZ* strain was comparable to the growth of bacteria exposed to the CuF produced by the wild-type strain at 48 h, thereby confirming that gliotoxin was the cause of bacterial growth inhibition (Fig. 1*A* and 1*B*). Because the CuF produced by the wild-type fungus at 48-hours induced the greatest increase in growth of *P. aeruginosa*, the material from this time point was chosen for further investigation.

**Fig. 2:**
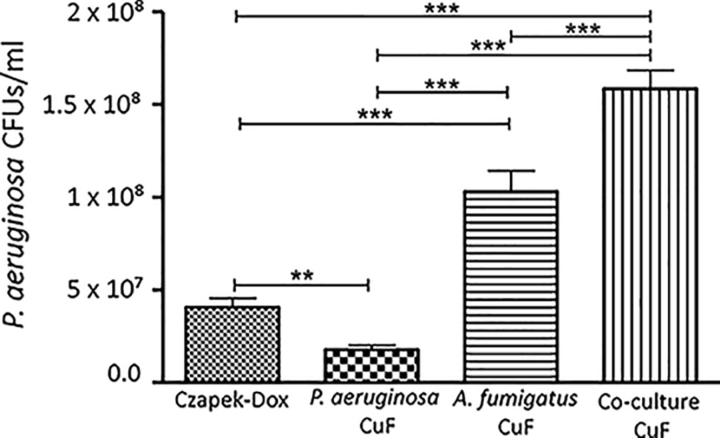
**Growth of *P. aeruginosa* (CFU/ml) cultured in Czapek-Dox media for 48 h compared with growth of *P. aeruginosa* cultured in Czapek-Dox media supplemented with *P. aeruginosa* CuF, *A. fumigatus* CuF and Co-culture CuF.** Changes in bacterial growth was greatest where *P. aeruginosa* was exposed to Co-culture CuF. ***: *p* < 0.001 **: *p* < 0.01 *: *p* < 0.05 ns: non-significant.

The effect on growth of *P. aeruginosa* when exposed to *A. fumigatus* CuF, was compared with that exposed to CuF produced in a co-culture of *A. fumigatus* and *P. aeruginosa* and with CuF produced by *P. aeruginosa*. To produce these CuFs, a 24-hour culture of bacteria was co-incubated with a 24-hour culture of *A. fumigatus* for a further 24 h so that the total incubation period was 48 h. Similarly, the 48-hour time point with which to allow *P. aeruginosa* to produce a CuF was chosen to maintain consistency with the time points at which the other CuFs were produced and had the greatest effect on bacterial growth. The growth of *P. aeruginosa* increased by 4-fold and 7-fold when cultured in Czapek-Dox supplemented with *A. fumigatus* CuF and Co-culture CuF respectively compared with that which was cultured in Czapek-Dox supplemented with *P. aeruginosa* CuF (Fig. 2).

To assess whether this growth effect was media-specific, *P. aeruginosa* was cultured for 24 h in minimal media supplemented with culture filtrates (CuF_MM_) produced in this media (*P. aeruginosa* CuF_MM_, *A. fumigatus* CuF_MM_ and Co-Culture CuF_MM_). No significant changes in growth were detected in bacteria cultured in the different CuF_MM_ (supplemental Fig. S3A). *P. aeruginosa* was cultured in an amino acid-rich defined medium, synthetic cystic fibrosis medium (SCFM), supplemented with *P. aeruginosa* CuF or Co-culture CuF produced in SCFM (CuF_SCFM_). No significant differences in growth were detected between *P. aeruginosa* exposed to Co-culture CuF_SCFM_ and *P. aeruginosa* CuF_SCFM_ (supplemental Fig. S3B).

##### Proteomic Analysis of A. fumigatus 48-hour Culture Filtrate

Mass spectrometry-based proteomics was performed on proteins collected from the 48 h-*A. fumigatus* CuF in order to investigate the components that may be promoting bacterial growth (supplemental Data set S1). The majority of proteins detected were enzymes associated with protein metabolism (*e.g.* l-amino acid oxidase, Tripeptidyl-peptidase sed2), cell wall biosynthesis (*e.g.* Protein ecm33, 1,3-beta-glucanosyltransferase Bgt1, Secreted beta-glucosidase sun1), gluconeogenesis (Triosephosphate isomerase, phosphoglucomutase PgmA, Glucose-6-phosphate isomerase, oxidative stress (*e.g.* Peroxiredoxin, Asp f3, Superoxide dismutase) and gliotoxin production (*e.g.* Thioredoxin reductase gliT, Cobalamin-independent methionine synthase). A number of proteins detected were involved in sugar metabolism (*e.g.* Beta-fructofuranosidase, Mannitol-1-phosphate 5-dehydrogenase, Glucooligosaccharide oxidase), and several proteins were associated with virulence (Alkaline protease 1, Alkaline protease 2, Aspergillopepsin-1, Major allergen Asp f 2). Additionally, several glucanases were detected in the culture filtrate (supplemental Data set S1).

##### A. fumigatus Generates An Amino Acid Rich Environment in Czapek-Dox

**Fig. 3:**
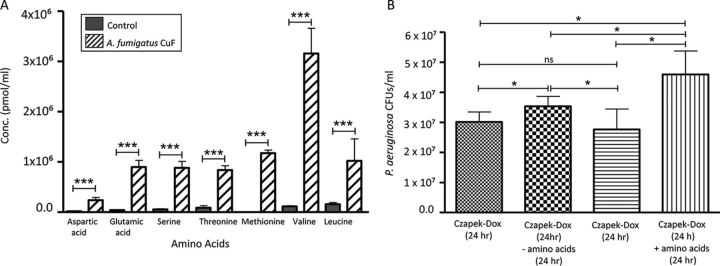
*A*, Protein hydrolysate analysis performed on *A. fumigatus* CuF produced at 48 h detected seven amino acids by RP-HPLC including aspartic acid, glutamic acid, serine, threonine, methionine, valine and leucine. *B*, *P. aeruginosa* (24-hour cultures) were exposed to un-supplemented Czapek-Dox or Czapek-Dox supplemented with the seven amino acids detected in the *A. fumigatus* CuF hyrolysates. *P. aeruginosa* growth (CFU/ml) increased by 1.6-fold when cultured in amino acid-supplemented Czapek-Dox for 24 h compared with that of bacteria exposed to un-supplemented (- amino acids) Czapek-Dox for 24 h, where growth increased by 1.17-fold.

Because Czapek-Dox does not contain amino acids in the original medium preparation, it was hypothesized the abundance of proteases and peptidases in the *A. fumigatus* CuF (supplemental Data set S1) was causing elevated levels of free amino acids or peptides in the culture filtrates and this was stimulating bacterial growth. The amino acid concentration in the culture filtrates was measured by the ninhydrin assay (supplemental Fig. S4). The ninhydrin test detected an amino acid concentration of 40 μg ±2.1/ml in the *A. fumigatus* CuF and 28 μg ±1.2/ml in the Co-culture CuF. Amino acids were not detected in the *P. aeruginosa* CuF or in the *A. fumigatus* CuF produced in MM which may explain why increased rates of bacterial growth were not observed in these media. To investigate the amino acid profile of the CuF, *A. fumigatus* CuFs were hydrolyzed and subjected to derivitization before being analyzed by RP-HPLC. Several amino acids were detected in the CuF including aspartic acid glutamic acid, serine threonine, methionine, valine, and leucine (Fig. 3*A*). To determine the role of such amino acids in driving bacterial growth, an overnight culture of *P. aeruginosa* was exposed to Czapek-Dox supplemented with the amino acids in the concentrations at which they were detected in the *A. fumigatus* CuF (aspartic acid (2.37 × 10^5^ ± 5.27 × 10^4^ pmol/ml), glutamic acid (8.99 × 10^5^ ± 1.3 × 10^5^ pmol/ml), serine (8.82 × 10^5^ ± 1.31 × 10^5^ pmol/ml), threonine (8.93 × 10^5^ ± 8.81 × 10^4^ pmol/ml), methionine (1.17 × 10^6^ ± 6.1 × 10^4^ pmol/ml), valine (1.14 × 10^5^ ± 1.37 × 10^4^ pmol/ml) and leucine (1.58 × 10^5^ ± 3.39 × 10^4^ pmol/ml). An increase in the growth of *P. aeruginosa* cultured in the amino acid-supplemented media compared with un-supplemented media indicate that amino acids in the media promote bacterial growth (Fig. 3*B*).

##### The Effect of A. fumigatus CuF and Co-culture CuF on the Proteome of P. aeruginosa

**Fig. 4:**
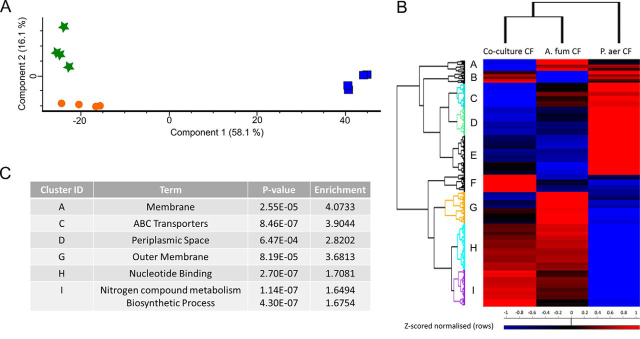
*A*, Principal component analysis (PCA) of *P. aeruginosa* exposed to Co-culture CuF (green) *A. fumigatus* CuF (orange) and *P. aeruginosa* CuF (blue). A clear distinction can be observed between each of the groups. *B*. Clusters based on protein-abundance profile similarities were resolved by hierarchical clustering of multi-sample comparisons between the three sample groups of *P. aeruginosa*. Nine clusters (A–I) were resolved comprising proteins that display similar expression profiles across treatments. Of these, six clusters (A, C, D, G–I) had statistically enriched Gene Ontology (GO) and KEGG terms associated with them (supplemental Data set S3) and the main terms are summarized for each in *C*.

Label free quantitative (LFQ) proteomics was employed to examine the whole cell proteomic response of *P. aeruginosa* exposed to *P. aeruginosa* CuF, *A. fumigatus* CuF or Co-culture CuF, and to investigate the proteins and pathways involved in regulating bacterial growth under these conditions. In total, 2317 proteins were initially identified, of which 1665 remained after filtering and processing (supplemental Data set S2A). Of the 1665 proteins identified post-imputation, 677 proteins in the *A. fumigatus* CuF-treated group (supplemental Data set S2B)and 611 proteins in the Co-culture CuF-treated group (supplemental Data set S2C) were determined to be statistically significant (*p* < 0.05) differentially abundant (SSDA) with a fold change of ± 2. A principal component analysis (PCA) was performed on all filtered proteins and identified distinct proteomic differences between the groups (Fig. 4*A*). Components 1 and 2 accounted for 71.3% of the total variance within the data, and all replicates resolved into their corresponding samples, with little variability between each sample. The groups exposed to *P. aeruginosa* CuF (control) displayed a clear divergence to those that were challenged with *A. fumigatus* CuF or Co-culture CuF. A distinct separation between the groups cultured in *A. fumigatus* CuF or Co-culture CuF was also observed.

Hierarchical clustering was performed on the Z-scored normalized LFQ intensity values for 1005 SSDA proteins identified (ANOVA; Benjamini Hochberg procedure, FDR cut-off value of ≤0.05). All four biological replicates resolved into their respective groups. Based on protein abundance profile similarities, nine protein clusters (A–I) were also resolved (Fig. 4*B*). GO and KEGG term enrichment analysis was performed on all protein clusters. Six clusters contained enriched terms (Cluster A, C, D, G–I; supplemental Data set S3B), with each cluster having a representative process or pathway characteristic to that group (Fig. 4*B* and 4*C*). Details of all clusters are included in the supplemental material (supplemental Data set S3A). Enriched terms contained in the clusters included membrane and integral to membrane (Cluster A), ABC transporters and periplasmic space (Cluster C), periplasmic space, (Cluster D), membrane and outer membrane proteins (Cluster G), nucleotide binding and transcription (Cluster H), cell division and amino acid metabolism (Cluster I).

**Fig. 5:**
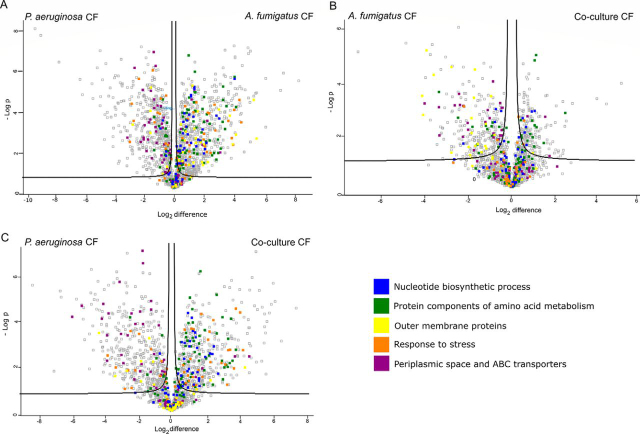
**Volcano plots derived from pairwise comparisons between *P. aeruginosa* cultured in (*A*) *A. fumigatus* CuF and *P. aeruginosa* CuF, (*B*) Co-culture CuF and *A. fumigatus* CuF and *C*) Co-culture CuF and *P. aeruginosa* CuF.** The distribution of quantified proteins according to the *p* value (- log10 *p* value) and fold change (log_2_ mean LFQ intensity difference) are shown. Proteins above the line are considered statistically significant (*p* value <0.05). Protein components of amino acid metabolism (green) and nucleotide biosynthetic process (blue) and response to stress (orange) are more abundant in bacteria cultured in *A. fumigatus* CuF and Co-culture CuF than in *P. aeruginosa* CuF. The relative abundance of proteins associated with the periplasmic space and ABC transporters (purple) was decreased in bacteria cultured in *A. fumigatus* CuF and Co-culture CuF compared with *P. aeruginosa* CuF. The relative abundance of outer membrane proteins (yellow) was greater in bacteria exposed to *A. fumigatus* CuF compared with *P. aeruginosa* CuF and lower in Co-culture CuF compared with *A. fumigatus* CuF and *P. aeruginosa* CuF.

Volcano plots were produced by pairwise Student's t-tests (*p* < 0.05) to determine the differences in protein abundance between two samples and to depict the changes in pathways and processes in which those proteins are involved (Fig. 5*A*–5*C*). The changes in biological pathways and processes observed in the volcano plots mirror the trends in the heat map (Fig. 4*B*).

The proteomic data arising from the Student's t-tests (*p* < 0.05) identified a significant increase in the relative abundance of proteins associated with cell division and cell wall formation in bacteria cultured in *A. fumigatus* CuF and Co-culture CuF, compared with *P. aeruginosa* CuF. There were no significant changes in these processes between bacteria cultured in *A. fumigatus* CuF and Co-culture CuF. The most abundant proteins associated with these processes are listed in supplemental Table 1A. The relative abundance of several components from the nucleotide biosynthesis pathway was increased in bacteria cultured in *A. fumigatus* CuF and Co-culture CuF compared with that exposed to *P. aeruginosa* CuF. SSDA proteins included in this pathway are listed in supplemental Table 1B and are highlighted in Fig. 5*A* and 5*B*. Student's t-tests performed on these groups, identified an increase in the relative abundance of SSDA proteins with the KEGG term “cellular amino acid biosynthesis” in bacteria exposed to *A. fumigatus* CuF and Co-culture CuF compared with *P. aeruginosa* CuF. This included the metabolism of valine, leucine, isoleucine, lysine, glutamine, cysteine, proline, serine and arginine (supplemental Table 1C, Fig. 5*A* and 5*B*).

In general, the relative abundance of proteins associated with ABC transporters were decreased in the bacteria exposed to *A. fumigatus* CuF compared with bacteria exposed to *P. aeruginosa* CuF (supplemental Table 1D). Most of these proteins were substrate binding proteins and ATP binding components of ABC transporters involved with the transport of amino acids, carbohydrates and lipids. Within this group, the three proteins with the greatest decrease in relative abundance were probable amino acid binding protein (PA3858; −17.1-fold), probable binding protein component of ABC iron transporter (PA5217; −38.6-fold) and binding protein component of ABC ribose transporter (rbsB; −57.7-fold). Of the 35 ABC-transport proteins listed, four were increased in abundance. Three of these, nitrate ABC transporter substrate-binding protein (nasS; +23.9-fold), probable ATP-binding component (PA4862; +13.2-fold) and molybdenum import ATP-binding protein (modC; +2.7-fold) are associated with the import of nitrate, urea and molybdate respectively. The full list of these proteins is included in supplemental Table 1D.

Pairwise *t*-tests identified increases and decreases in the relative abundance of proteins associated with a stress response and DNA damage repair in *P. aeruginosa* exposed to *A. fumigatus* CuF and Co-culture CuF compared with bacteria exposed to *P. aeruginosa* CuF (Table I). Xenobiotic reductase B (Xen B), Lon protease (lon), Alkyl hydroperoxide reductase subunit F (ahpF) and Bifunctional enzyme CysN/CysC (CysNC) showed the greatest fold-change in *P. aeruginosa* exposed to *A. fumigatus* CuF and Co-culture CuF compared with that which was exposed to *P. aeruginosa* CuF. Except for Xen B, there were no significant changes in the relative abundance of these proteins between the groups exposed to *A. fumigatus* CuF and Co-culture CuF. Catalase HPII (katE), magD (magD) and cold-shock protein (CspD) showed the greatest decrease in relative abundance in bacteria exposed to *A. fumigatus* CuF and Co-culture CuF compared with *P. aeruginosa* CuF-exposed groups.

**Table I:** Pathways that are most affected by bacterial culture conditions. Differential expression of proteins associated with a stress response and DNA damage repair, respiration and outer membrane proteins in P. aeruginosa: statistically significant proteins and associated relative fold difference arising from pairwise Student's t-tests (p < 0.05) between P. aeruginosa exposed to A. fumigatus CuF and P. aeruginosa CuF, Co-culture CuF and P. aeruginosa CuF and Co-culture CuF and A. fumigatus CuF. A.f.; A. fumigatus CuF, P.a.; P. aeruginosa CuF, Cc; Co-culture CuF. n.s. indicates that the relative fold difference was non-significant

Pathway	Gene	Protein	Relative fold difference		
			A.f. v P.a.	Cc v P.a.	Cc v A.f.
Stress response	xenB	Xenobiotic reductase B	304.4	156	−1.9
	lon	Lon protease	14.2	18.6	ns
	ahpF	Alkyl hydroperoxide reductase subunit F	20	16.5	ns
	cysNC	Bifunctional enzyme CysN/CysC	15.4	13.8	ns
	uvrB	UvrABC system protein B	15.1	12.6	ns
	cysI	Sulfite reductase	12.2	10	ns
	recA	Protein RecA	5.35	7.6	ns
	trxB2	Thioredoxin reductase	6.5	5.5	ns
	polA	DNA polymerase I	6.8	4.9	ns
	ppx	Exopolyphosphatase	5.1	4.7	ns
	ruvB	Holliday junction ATP-dependent DNA helicase RuvB	4.5	3.5	ns
	katE	Catalase HPII	−6.3	−13.6	ns
	magD	magD	−3	−12.2	ns
	cspD	Cold-shock protein CspD	−7.6	−9.7	ns
	msrB	Peptide methionine sulfoxide reductase	−7	−5.3	ns
	SenC	SenC	ns	−5	ns
	osmC	Osmotically inducible protein OsmC	−5	−4.3	ns
	phoB	Phosphate regulon transcriptional regulator	ns	−3.8	ns
	PA0838	Glutathione peroxidase	−2.1	−2.3	ns
	gor	Glutathione reductase	−2	−1.7	ns
	lexA	LexA repressor	−1.4	−1.6	ns
Respiration	PA5190	Probable nitroreductase	38.8	58.5	1.5
	narG	Respiratory nitrate reductase alpha chain	30.3	53.8	ns
	ccoO2	Cytochrome c oxidase, cbb3-type, CcoO subunit	20.53	48.5	2.4
	moaB1	Molybdenum cofactor biosynthesis protein B	21.3	43.2	ns
	nosZ	Nitrous-oxide reductase	20.54	39	ns
	hemN	Oxygen-independent coproporphyrinogen-III oxidase	14.7	29.7	2
	nirB	Assimilatory nitrite reductase large subunit	6.6	25.6	3.9
	narH	Respiratory nitrate reductase beta chain	9.5	19.3	ns
	nirD	Assimilatory nitrite reductase small subunit	Ns	11.2	3.9
	moeA1	Molybdopterin molybdenum transferase	5.6	8.98	ns
	exaB	Cytochrome c550	−447.6	−286.3	ns
	exaA	Quinoprotein alcohol dehydrogenase (cytochrome c)	−13	−47.6	−3.7
	PA2171	Hemerythrin-domain containing protein	−73.9	−90.5	ns
	napA	Periplasmic nitrate reductase	−7	−34.8	−5
	napB	Periplasmic nitrate reductase, electron transfer subunit	−9.9	−5.83	ns
	bkdB	Lipoamide acyltransferase	−2.7	−24.1	−9
	bkdA1	2-oxoisovalerate dehydrogenase subunit alpha	Ns	−4.9	−5.2
	pdhA	Pyruvate dehydrogenase E1 component subunit alpha	−16.4	−7	−2.4
	PA3415	Dihydrolipoamide acetyltransferase	−3.4	−16.4	−4.7
	lpdV	Dihydrolipoyl dehydrogenase	−1.9	15.4	−8.2
Outer membrane	MexE	RND multidrug efflux membrane fusion protein	132.3	47.27	ns
	oprC	Outer membrane efflux protein OprC	38.6	ns	−15.7
	oprG	Outer membrane protein OprG	17.3	4.9	−3.5
	oprM	Outer membrane protein OprM	15	ns	−16.3
	MexF	Efflux pump membrane transporter	10.2	ns	ns
	oprE	Anaerobically-induced outer membrane porin OprE	3.2	ns	−3.1
	oprJ	Outer membrane protein OprJ	2.7	ns	−3.2
	oprO	Porin O	2.45	ns	−2.9
	opr86	Outer membrane protein assembly factor BamA	2.3	ns	ns
	opmH	Channel protein TolC	2	ns	−1.9
	MexC	RND multidrug efflux membrane fusion protein	1.6	ns	−3.1
	oprF	Outer membrane porin F	1.2	ns	ns
	oprQ	Outer membrane protein OprQ	−2.6	−23.5	−9.1
	opdQ	Outer membrane porin OpdQ	−2.1	−13.7	−6.5
	oprD	Porin D	−9.7	−5.6	ns
	oprI	Major outer membrane lipoprotein	−6.6	−3.5	ns
	oprH	PhoP/Q & low Mg2+ inducible outer membrane protein	ns	−3.5	−3.8
	MexB	Multidrug resistance protein MexB	−2.28	ns	ns

The proteomic data arising from the Student's t-tests (*p* < 0.05) identified a significant increase in the abundance of proteins associated with anaerobic respiration, including denitrification enzymes, and a decrease in the levels of proteins associated with aerobic respiration in bacteria exposed to Co-culture CuF and *A. fumigatus* CuF compared with that exposed to *P. aeruginosa* CuF. Functionally annotated SSDA proteins with the greatest differential increase or decrease in abundance involved in anaerobic or aerobic respiration respectively, are listed in Table I. Student's t-tests on the post-imputation data set revealed distinct changes in the relative abundance of outer membrane proteins (OMPs) and efflux pumps between *P. aeruginosa* cultures exposed to *A. fumigatus* CuF and *P. aeruginosa* CuF, Co-culture CuF and *P. aeruginosa* CuF and Co-culture CuF and *A. fumigatus* CuF. There was a clear increase in the number of, and the relative abundance of several OMPs in *P. aeruginosa* exposed to *A. fumigatus* CuF compared with the other groups (Table I, Fig. 5*C*).

## DISCUSSION

The clinical importance of co-infection with *A. fumigatus* and *P. aeruginosa* in the cystic fibrosis lung has been highlighted in several reports because of its association with deteriorating lung function (8, 9, 11). Despite the ability of *A. fumigatus* to survive and persist in the hostile environment of the cystic fibrosis lung, *P. aeruginosa* predominates as the primary pathogen and is the leading cause of poor patient outcome (8, 27). The objective of the current study was to characterize the factors that enable *P. aeruginosa* to outcompete *A. fumigatus.*

The sputum of individuals with cystic fibrosis is characterized by high nitrate content and is thus conducive to the growth of denitrifying microorganisms such as *P. aeruginosa* (28, 29). Czapek-Dox is a defined synthetic media used for the cultivation of microorganisms that can utilize sodium nitrate as the only source of nitrogen and provides a nutrient-limiting, nitrogen-rich environment. Because of the poor nutritional content, it is frequently used for inducing the production of fungal secondary metabolites, such as fumagillin and gliotoxin (30, 31). Although cultivation of *P. aeruginosa* in Czapek-Dox is possible, *A. fumigatus* grows faster in this media. As such, we sought to investigate how culture filtrates produced by *A. fumigatus* in Czapek-Dox would affect the growth and proteome of *P. aeruginosa*. The results presented here demonstrate a role for *A. fumigatus* in promoting the growth of *P. aeruginosa* by altering the environment.

We observed a significant increase in bacterial growth when exposed to *A. fumigatus* culture filtrates (CuF) produced in Czapek-Dox after 48 h. *A. fumigatus* CuF produced at 96 h inhibited bacterial growth. To investigate this finding further, the *A. fumigatus* CuFs were analyzed for gliotoxin, which has been reported to have anti-*P. aeruginosa* activity (20). RP-HPLC analysis determined that gliotoxin concentrations were highest in the 96-hour CuF, which may explain why *P. aeruginosa* growth was inhibited at this time point. To confirm that gliotoxin was the source of growth inhibition, *P. aeruginosa* was exposed to *A. fumigatus* CuF produced by a gliotoxin mutant lacking the GliZ gene (Δ*gliZ*). GliZ is indispensable for gliotoxin production (32) and gliotoxin was undetectable by RP-HPLC from the 96-hour CuF produced by the fungal strains in Czapek-Dox. Bacterial growth was greater in cultures exposed to the 96-hour CuF produced by Δ*gliZ* strains, indicating that gliotoxin was the cause of *P. aeruginosa* growth inhibition in the CuF produced by the wild-type strain.

Qualitative mass spectrometry-based proteomics was used to identify the contents of the 48-hour *A. fumigatus* CuF with a view to characterizing the secreted fungal products that may be influencing the growth of *P. aeruginosa*. A large proportion of the proteins detected were secreted enzymes associated with peptide metabolism, *i.e.* proteases and peptidases, including tripeptidyl-peptidase sed2, alkaline protease 1 and aspergillopepsin-1 (33, 34, 35, 36). *A. fumigatus* assimilates exogenous amino acids via membrane transporters and thus secretes a range of proteases to digest larger peptides and proteins prior to import into the cell (37). In the cystic fibrosis lung, secreted proteins are major contributors to allergic responses and virulence in ABPA (37, 38, 39). These include Aspergillopepsin, alkaline protease and Asp f 2 and Asp f 4 proteases. Analysis of cystic fibrosis sputum has shown that it contains higher concentrations of amino acids than control sputum (40, 41). Synthetic cystic fibrosis medium (SCFM) contains an amino acid content comparable to that found in the sputum of cystic fibrosis patients (21). In this study, the growth rate of *P. aeruginosa* was significantly greater when cultured in sterile SCFM, a growth medium rich in amino acids, than that in Czapek-Dox, suggesting that the amino acid content of the culture media influences the rate of bacterial growth.

Qualitative proteomic analysis of the 48-hour *A. fumigatus* CuF identified an abundance of peptide-metabolizing enzymes, indicating the presence of amino acids in the liquid medium. This was confirmed by the ninhydrin test, which detected free amino acids in the *A. fumigatus* CuF and in the Co-culture CuF. Amino acids were not detected in the *P. aeruginosa* CuF or in any culture filtrates produced in MM. This may explain why the same rate of bacterial growth observed in *A. fumigatus* and Co-culture CuF was not observed in *P. aeruginosa* CuF or in culture filtrates produced in MM. The amino acid profile of the *A. fumigatus* CuF was further analyzed by RP-HPLC. A range of amino acids were identified by this method, including aspartic acid, glutamic acid, serine, threonine, methionine and the branched-chain amino acids, valine and leucine. These results indicate that *A. fumigatus* creates an amino acid-rich environment in which *P. aeruginosa* can proliferate, possibly by metabolizing the medium to produce substrates more easily assimilated by the bacteria.

Label-free quantitative (LFQ) proteomics involves the simultaneous identification and quantification of thousands of proteins (the ultimate determinants of phenotypes) from a single sample. This approach has previously been employed to characterize the *P. aeruginosa* proteome in response to iron limiting conditions (42). In this study, LFQ proteomics was utilized to investigate the determinant mechanisms that enable *P. aeruginosa* to proliferate in an environment containing *A. fumigatus* secondary metabolites, degradative enzymes, and limited nutrients.

The proliferation of growth observed in *P. aeruginosa* exposed to *A. fumigatus* CuF and Co-culture CuF compared with *P. aeruginosa* CuF was reflected by an increase in the relative abundance of proteins associated with DNA replication (DNA gyrase subunit B and Ribonucleoside-diphosphate reductase), cell division (*e.g.* Cell division protein FtsZ and ZapE) and cell wall biosynthesis (Mur enzymes).

There were significant changes in the abundance of proteins associated with the metabolism and transport of amino acids in bacteria exposed to *A. fumigatus* CuF and Co-culture CuF compared with those exposed to *P. aeruginosa* CuF. In addition to its requirement for growth, amino acid metabolism in *P. aeruginosa* is necessary for the intracellular synthesis of quorum sensing (QS) molecules which mediate cell-cell communication in a cell density-dependent manner and for the biosynthesis of secondary metabolites such as phenazine-1-carboxylic acid and pyocyanin (40, 43, 44).

The proteomic data identified several ATP-binding cassette (ABC) transporters involved with the transport of amino acids detected in the *A. fumigatus* CuF by RP-HPLC, including branched-chain amino acids, glutamate, aspartate, and methionine. The relative abundance of proteins associated with ABC transporters was significantly less in *P. aeruginosa* grown in Co-culture CuF and *A. fumigatus* CuF compared with bacteria cultured in *P. aeruginosa* CuF. Most of these ABC transporters were importers associated with the import of nutrients such as amino acids and carbohydrates into the cell. The import of substrates is tightly regulated and the expression of ABC transporters is increased or decreased depending on the nutrient availability and nutritional requirements of the organism (45, 46). A decrease in the relative abundance of amino acid ABC transporters in bacteria exposed to Co-culture CuF and *A. fumigatus* CuF compared with *P. aeruginosa* CuF indicate a greater need for uptake of nutrients from the environment in *P. aeruginosa* cells exposed to the latter. This suggests the availability of nutrients in the CuF produced by *A. fumigatus* was greater than that generated by *P. aeruginosa* in Czapek-Dox.

Interestingly, ABC transporters involved in the uptake of nitrate and molybdate were two of the four ABC transporters increased in *P. aeruginosa* exposed to Co-culture CuF. The increase in nitrate and molybdate ABC transporters in *P. aeruginosa* exposed to Co-culture CuF indicate anaerobic growth. Molybdate is required for the formation of molybdoenzymes such as nitrate reductases, which are essential for the use of nitrates during anaerobic respiration (47), so much so, that mutants for molybdate ABC transporters (ModABC) are unable to grow anaerobically and are less virulent under aerobic and anaerobic conditions (48).

*P. aeruginosa* is a facultative anaerobe and can perform denitrification under anaerobic conditions and in the presence of nitrate (49). The reduction of nitrate to nitrite by nitrate reductases contributes to energy production more than nitrite reduction (50, 51). Membrane nitrate reductases (NarGHI) are essential for anaerobic growth and are expressed at the expense of periplasmic nitrate reductases (NapAB) (51). Our data identified a decrease in the relative abundance of NapA and NapB in bacteria grown in *A. fumigatus* CuF and Co-culture CuF compared with *P. aeruginosa* CuF, and an increase in the relative abundance of NarG and NarH, the levels of which were highest in *P. aeruginosa* grown in Co-culture CuF. The relative abundance of the assimilatory nitrite reductases NirB and NirD, which catalyze the reduction of nitrite to ammonium (50), were greatest in bacteria exposed to Co-culture CuF. Taken together, these data suggest that co-incubation of *A. fumigatus* and *P. aeruginosa* create a nitrate-rich environment under which *P. aeruginosa* can adapt by upregulating the denitrification pathway.

The cystic fibrosis airway is characterized by low oxygen availability and high nitrate content because of oxygen consumption by pro-inflammatory immune cells (49). Cystic fibrosis sputum contains levels of nitrate sufficient to support anaerobic growth of *P. aeruginosa* and membrane nitrate reductases are essential for bacterial survival under these levels of nitrates (49, 52). High levels of nitric oxide (resulting from denitrification) can result in nitrosative stress which *P. aeruginosa* counteracts through a variety of detoxification systems (53). Among the most abundant proteins detected in *P. aeruginosa* exposed *A. fumigatus* CuF and Co-culture CuF were the RND family efflux membrane protein MexE and xenobiotic reductase (XenB). These proteins are induced by nitrosative stress thereby suggesting a defensive mechanism against high NO levels that occur during denitrification (54, 55).

The proteomic results identified an increase in the relative abundance of several other protective enzymes in *P. aeruginosa* exposed to *A. fumigatus* CuF and Co-culture CuF, including lon protease and Alkyl hydroperoxide reductase subunit F (AhpF). Lon protease regulates biofilm formation, motility and is essential for virulence (56, 57). AhpF is associated with detoxification of benzene derivatives and increased tolerance to zinc-containing compounds (58).

Differences in the relative abundance of outer membrane proteins (OMPS) were identified in bacteria exposed to *A. fumigatus* CuF compared with that of Co-culture CuF. This suggests alterations in the regulatory responses to the environment created by *A. fumigatus* in the absence or presence of *P. aeruginosa*. OMPs regulate the influx and efflux of nutrients and potentially toxic compounds in and out of the cell. The greatest changes in the relative abundance of OMP were observed in OprC, OprG and OprM. OprC regulates the influx of copper ions into the cell (59, 60). OprG is up-regulated under anaerobic conditions and is associated with amino acid import (61, 62). OprM forms the ejection component of the MexAB-OprM efflux system and is responsible for resistance against β-lactams and quinolones, among others (63, 64). In *P. aeruginosa* cultures exposed to *A. fumigatus* CuF or Co-culture CuF, the relative abundance of OprD was decreased. Down-regulation of this protein has been associated with resistance to carbapenems (65). In general, the increase in OMP expression indicates a greater need to expel potentially potent compounds from the bacterial cell exposed to *A. fumigatus* CuF and perhaps suggests a less toxic secretome in the medium in which *A. fumigatus* had been co-cultured with *P. aeruginosa*. These data highlight some changes that occur in the proteomic profile of *P. aeruginosa* upon exposure to the CuF produced by *A. fumigatus* in the presence or absence of the bacteria. This may be important in the context of infection as the bacterial phenotype, which is dictated in part by its environment, plays a critical role in the outcome for the host (7, 66).

## CONCLUSION

This study demonstrates that the presence of *A. fumigatus* has the potential to stimulate *P. aeruginosa* growth and pathogenesis by creating an environment that promotes bacterial growth, a result which, in the context of cystic fibrosis, has important clinical relevance, as co-infection with these pathogens is associated with poor patient outcome (8, 11). We employed LFQ proteomics to characterize the response of *P. aeruginosa* to an environment previously inhabited by *A. fumigatus*. We reveal that *A. fumigatus* altered this environment to favor the growth of, and confer fitness to *P. aeruginosa* under conditions known to exist in the cystic fibrosis airways, such as high nitrate and amino acid content. This is the first study that examines the whole-cell proteomic response of *P. aeruginosa* under these conditions. The results presented here can be used to understand the dynamics that occur between *P. aeruginosa* and *A. fumigatus* at a molecular level. Understanding the strategies that permit *P. aeruginosa* to dominate as the primary pathogen in the cystic fibrosis lung is critical for the identification of potential targets for therapeutic interventions.

## DATA AVAILABILITY

Proteomic data can be accessed in the PRIDE partner repository with the data set identifiers: PXD015056 (LFQ proteomic data of *P. eruginosa* proteome) and PXD018112 (Qualitative proteomic data of *A. fumigatus* culture filtrates).
